# Functions of Fun30 Chromatin Remodeler in Regulating Cellular Resistance to Genotoxic Stress

**DOI:** 10.1371/journal.pone.0121341

**Published:** 2015-03-25

**Authors:** Xin Bi, Qun Yu, Jasmine Siler, Chong Li, Ali Khan

**Affiliations:** Department of Biology, University of Rochester, Rochester, New York, United States of America; Dana-Farber/Harvard Cancer Institute, UNITED STATES

## Abstract

The *Saccharomyces cerevisiae* Fun30 chromatin remodeler has recently been shown to facilitate long-range resection of DNA double strand break (DSB) ends, which proceeds homologous recombination (HR). This is believed to underlie the role of Fun30 in promoting cellular resistance to DSB inducing agent camptothecin. We show here that Fun30 also contributes to cellular resistance to genotoxins methyl methanesulfonate (MMS) and hydroxyurea (HU) that can stall the progression of DNA replication. We present evidence implicating DNA end resection in Fun30-dependent MMS-resistance. On the other hand, we show that Fun30 deletion suppresses the MMS- and HU-sensitivity of cells lacking the Rad5/Mms2/Ubc13-dependent error-free DNA damage tolerance mechanism. This suppression is not the result of a reduction in DNA end resection, and is dependent on the key HR component Rad51. We further show that Fun30 negatively regulates the recovery of *rad5Δ* mutant from MMS induced G2/M arrest. Therefore, Fun30 has two functions in DNA damage repair: one is the promotion of cellular resistance to genotoxic stress by aiding in DNA end resection, and the other is the negative regulation of a Rad51-dependent, DNA end resection-independent mechanism for countering replicative stress. The latter becomes manifest when Rad5 dependent DNA damage tolerance is impaired. In addition, we find that the putative ubiquitin-binding CUE domain of Fun30 serves to restrict the ability of Fun30 to hinder MMS- and HU-tolerance in the absence of Rad5.

## Introduction

Genotoxic agents may cause DNA damage and/or block DNA replication leading to genome instability, and cells have evolved a variety of mechanisms to counter the effects of these agents on DNA. These mechanisms include DNA damage repair and tolerance/bypass pathways as well as DNA damage checkpoints [1]. DNA double-strand breaks (DSBs) can be repaired by homologous recombination (HR) or non-homologous end joining (NHEJ), and single-strand DNA damages are subject to repair by nucleotide excision repair (NER), base excision repair (BER), or DNA mismatch repair (MMR) [2–6]. In S phase of the cell cycle, base modifications can stall the DNA polymerase. DNA damage tolerance (DDT) (also know as DNA damage bypass, DDB, and post-replication repair, PRR) pathways enable DNA replication to pass through the damaged bases without repairing them, thereby allowing the completion of DNA replication, and leaving the damages to be repaired after DNA replication [7,8].

Methyl methanesulfonate (MMS), hydroxyurea (HU) and camptothecin (CPT) are widely used genotoxins in DNA repair studies that impact the genome via different mechanisms. MMS is an alkylating agent that predominantly methylates G and A bases in DNA [9]. MMS-induced DNA methylation can block the progression of the DNA replication fork. HU inhibits ribonucleotide reductase [10], resulting in the depletion of dNTP pools, thereby slowing/halting replication fork progression. Stalling DNA replication by MMS or HU leads to single stranded DNA (ssDNA) gaps at the replication fork due to the uncoupling of DNA polymerase and helicase activities, and may potentially cause the collapse/breakage of arrested forks, generating DSBs [11–13]. CPT is a specific inhibitor of DNA topoisomerase I (Topo I) that binds Topo I covalently linked to nicked DNA, and prevents DNA religation [14,15]. In S phase, the collision between the DNA replication fork and the CPT-Topo I-DNA ternary complex results in DSB [16]. A given DNA repair or bypass pathway may or may not be able to repair the damage caused by a specific genotoxin depending on the nature of the DNA damage (e.g., [17,18]).

Since eukaryotic DNA is packed into chromatin, response to DNA damage occurs in the context of chromatin. Accordingly, chromatin structure and factors involved in chromatin assembly, modification and remodeling may participate directly or indirectly in DNA damage response [19,20]. For example, DSB repair involves certain histone modifications, histone variants, as well as chromatin remodeling activities [21]. The *Saccharomyces cerevisiae* Fun30 protein is a recently characterized chromatin remodeler that plays a role in cellular resistance to CPT [22]. It has been shown that Fun30 is recruited to DSBs and facilitates the resection of DSB ends, which is believed to aid in subsequent HR repair [23–25]. Fun30 is thought to overcome the chromatin barrier to DSB end resection by remodeling nucleosomes [23–25].

Interestingly, previous studies found *FUN30* deletion (*fun30Δ*) not to affect cellular resistance to MMS and HU [22,24], despite that these agents can potentially lead to DSBs as does CPT. We reasoned that the outcome of a genotoxin-sensitivity test might be influenced by genotoxin dosage and sometimes the genetic background of the strain used. To test this possibility, we systematically examined the effects of *fun30Δ* on the resistance to MMS, HU or CPT at different concentrations of a series of yeast strains derived originally from the commonly used S228c and W303 strains [26]. We found that Fun30 deletion reduces cellular resistance to MMS and HU at relatively high concentrations. We also found that Fun30 negatively regulates the tolerance to MMS and HU of cells lacking the Rad5/Mms2/Ubc13 mediated DDT pathway. These results revealed novel functions of Fun30 chromatin remodeler in cellular response to genotoxic stress.

## Materials and Methods

### Plasmids and yeast strains

Plasmid pRAD5 was made by replacing the NgoMIV-XhoI fragment of pRS415 [27] with a fragment consisting of the *RAD5* open reading frame (ORF) plus its 500 bp upstream and 340 bp downstream sequences. Plasmids pRS424-EXO1 and pRS424-exo1 are identical to pSM502 and pSM638, respectively, and have been previously described [28]. Briefly, pSM502 contains the *EXO1* gene on the high copy 2μ vector pRS424, whereas pSM638 contains a mutant *EXO1* allele coding for Exo1 with a D173A mutation that disrupts Exo1 activity [28]. Plasmid pRS414-FUN30 was made by inserting the NgoMIV-*FUN30*-AlwNI fragment from plasmid pLF230 [29] into plasmid pRS414 [27]. The CUE domain of *FUN30* (coordinates 228 to 333) was deleted from pRS414-FUN30 to make pRS414-FUN30-CUEΔ. Nucleotides 244-TT-245 of *FUN30* open reading frame on pRS414-FUN30 were changed to GC, and C247 to G by site-directed mutagenesis to make pRS414-FUN30-CUE** encoding Fun30 with its conserved phenylalanine 82 and proline 83 in the CUE motif replaced by alanines. Nucleotide A1608 of *FUN30* on pRS414-FUN30 was changed to G to make pRS414-FUN30-K603R encoding Fun30-K603R. Changing the conserved lysine 603 in the ATPase domain of Fun30 to arginine inactivates the enzymatic activity [22].

Yeast strains are listed in S1 Table. Strain YXB726 was derived from YXB102 [30] by replacing its *URA3*-marked p*CEN-URA3-HHF1* plasmid with the *ADE2*-marked plasmid pRS412-*HHT2-HHF2*, followed by inserting *URA3* into the *HML* locus and replacing *HHT1* with *LoxP*. This strain was made originally for the purpose of simultaneously examining the effects of histone H4 mutations on genotoxin resistance and *HML* silencing. Gene knockout was achieved by replacing the ORF of the gene of interest with *KanMX*, *NatMX* or *ble2* cassette, or *TRP1*. Relevant genotypes of the strains were confirmed by Southern blotting. YXB013-5 and -6 were made by transforming plasmid pRS415 into CCFY101 and YXB013-3, respectively. YXB13-7 and -8 were made by transforming plasmid pRAD5 into YXB101 and YXB013-3, respectively. YXB013-96 through -100 were made by transforming plasmids pRS414, pRS414-FUN30, pRS414-FUN30-CUEΔ, pRS414-FUN30-CUE** and pRS414-FUN30-K603R into YQY726, respectively. YXB013-101 through -105 were similarly derived from YXB013-2.

### Quantitative infrared western analysis of PCNA modifications

About 4 x 10^8^ exponentially growing cells were collected and resuspended in 200 μl of 20% trichloroacetic acid (TCA) solution. Cells in TCA solution were lysed by the glass bead method. Proteins were precipitated and resuspended in 200 μl of Laemmli buffer, and neutralized by 100 μl of 1 M Tris, pH 8.8. The sample was then boiled for 3 min, and 10 mg of proteins was subjected to 12% SDS-PAGE and wetsern blotting. The blot was probed with an rabbit anti-PCNA antibody in Odyssey blocking buffer (LI-COR Biosciences), and then with IRDye 800CW goat-anti-rabbit secondary antibody (LI-COR Biosciences) and imaged using the LI-COR Odyssey CLx Infrared Imaging System (LI-COR Biosciences). Quantification was done with Image Studio Lite software (LI-COR Biosciences).

### Fluorescence activated cell sorting

Cells for fluorescence activated cell sorting (FACS) analysis were grown at 30°C to log phase (OD_600_ = 0.6) and then divided into two aliquots. One aliquot was treated with 0.003% MMS for 4 hr with shaking, and the other was mock treated. After MMS exposure, cultures of strains 45 and 46 were treated with 5% sodium thiosulfate to deactivate MMS, and resuspended in fresh medium for further incubation. Samples for FACS were prepared as described [31] and analyzed on a FACSCalibur (Becton, Dickinson and Company). Data acquisition was performed using the FlowJo software.

## Results

### Fun30 contributes to cellular tolerance to genotoxins that stall the progression of DNA replication

**Fig 1:**
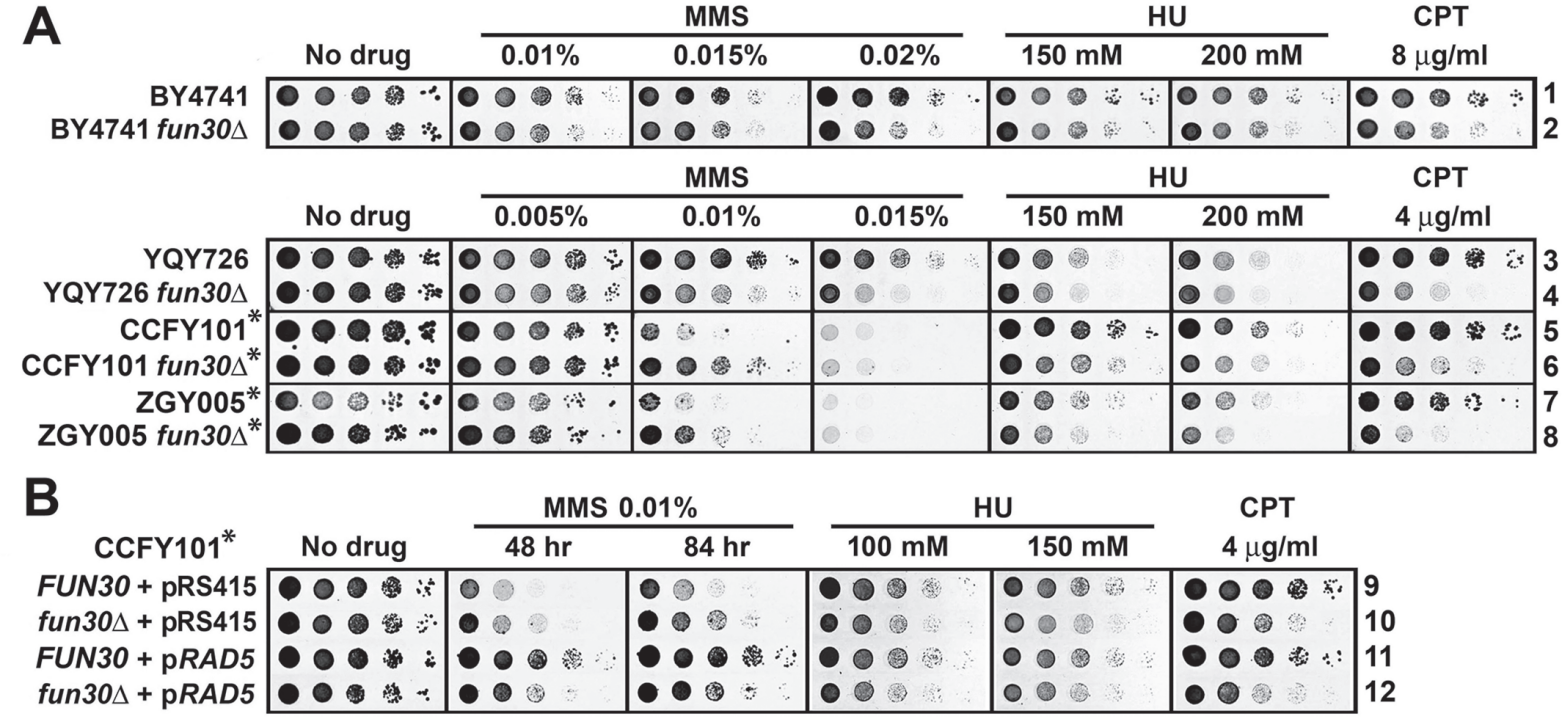
Fun30 contributes to cellular tolerance to genotoxins MMS, HU and CPT. Shown are growth phenotypes of strains 1–12 (S1 Table) on indicated media. Strains bearing the *rad5-535* mutation are marked with asterisks. Cells were grown to late log phase and serial 10-fold dilutions were spotted on SC (synthetic complete medium) with or without MMS, HU or CPT. The plates were incubated for 3 days unless otherwise indicated.

We reexamined whether Fun30 plays roles in cellular resistance to MMS and HU by monitoring the effect of *fun30Δ* on the growth of a series of strains derived originally from S228c or W303 in the presence of different dosages of MMS or HU. We found that *fun30Δ* decreased the resistance to HU (at a relatively high concentration of 150 mM or 200 mM) of all strains (Fig. 1A, compare strains 2, 4, 6, 8 with 1, 3, 5, and 7, respectively, on HU-containing medium). However, surprisingly, *fun30Δ* appeared to have varying effects on MMS resistance depending on the strains tested. Specifically, *fun30Δ* made strains BY4741 and YQY726 more sensitive to MMS, but rendered CCFY101 and ZGY005 more resistant to MMS at a relatively high concentration (0.01%) (Fig. 1A, compare strains 2, 4, 6, 8 with 1, 3, 5, and 7, respectively on MMS-containing medium). We noticed that BY4741 and YQY726 were generally more resistant to MMS than CCFY101 and ZGY005 (Fig. 1A). These results demonstrated again that the genetic background of a strain may affect its sensitivity to genotoxins, and may even determine if a factor plays a positive or negative role in DNA damage response. Consistent with previous studies, we found that *fun30Δ* reduced cellular tolerance to CPT to various degrees in all strains tested (Fig. 1A).

An investigation of the pedigrees of the strains used in the above tests revealed that BY4741 and YQY726 were derived from S288c, and CCFY101 and ZGY005 from W303. Therefore, it is possible that a genetic difference between S288c and W303 was responsible for their opposite modes of response to MMS treatment in the absence of Fun30. The original W303 strain differs from S288c in part by bearing a mutation in the *RAD5* gene (*rad5-535)* resulting in a change from arginine to glycine at position 535 in Rad5 protein [32]. We found that both CCFY101 and ZGY005 strains retained the *rad5-535* mutation (S1 Fig.). On the other hand, S288c derivatives BY4741 and YQY726 did not have the *rad5-535* mutation (S1 Fig.). Given the fact that Rad5 plays a major role in DNA damage tolerance [33], it was not surprising that CCFY101 and ZGY005 were more sensitive to MMS than BY4741 and YQY726 (Fig. 1A).

The fact that *fun30Δ* reduced MMS resistance of *RAD5* strains BY4741 and YQY726, but made *rad5-535* strains CCF101 and ZGY005 more resistant to MMS led us to propose that Fun30 facilitates cellular resistance to MMS when *RAD5* is intact, but plays an inhibitory role in MMS-resistance in the presence of the *rad5-535* mutation. To test this hypothesis, we introduced a plasmid bearing the intact *RAD5* gene (pRAD5) or the vector (pRS415) into CCFY101 and its *fun30Δ* derivative. The presence of pRS415 had no effect on the resistance of these strains to MMS, HU or CPT (Fig. 1, compare 9 and 10 with 5 and 6, respectively). On the other hand, pRAD5 made CCFY101 markedly more resistant to MMS, but had no effect on CCFY101’s resistance to HU and CPT (Fig. 1B, compare 11 with 9). This result indicates that the reduced tolerance to MMS of CCF101 compared to BY4741 or YQY726 is attributable to the *rad5-535* mutation, and that *RAD5* is dominant over *rad5-535*. Importantly, *fun30Δ* significantly reduced MMS tolerance of CCFY101 bearing pRAD5 (Fig. 1B, compare 12 with 11). Therefore, CCFY101 supplemented with an ectopic copy of *RAD5* behaves similarly to *RAD5* strains BY4741 and YQY726 with respect to the impact of *fun30Δ* on MMS tolerance (Fig. 1, compare 11 and 12 with 1 through 4). This supports the aforementioned notion that Fun30 plays a positive role in MMS resistance when *RAD5* is intact, but becomes inhibitory in the presence of the *rad5-535* mutation. In contrast, *fun30Δ* reduced cellular tolerance to CPT and HU in both *RAD5* and *rad5-535* backgrounds (Fig. 1). Therefore, *rad5-535* seems to specifically affect how Fun30 functions in cellular response to MMS. Taken together, the above results demonstrate that Fun30 plays roles in cellular resistance to genotoxins MMS, HU and CPT, and its function in MMS-tolerance is influenced by Rad5.

### *EXO1* is a high copy suppressor of MMS-sensitivity of *fun30Δ* mutant

**Fig 2:**
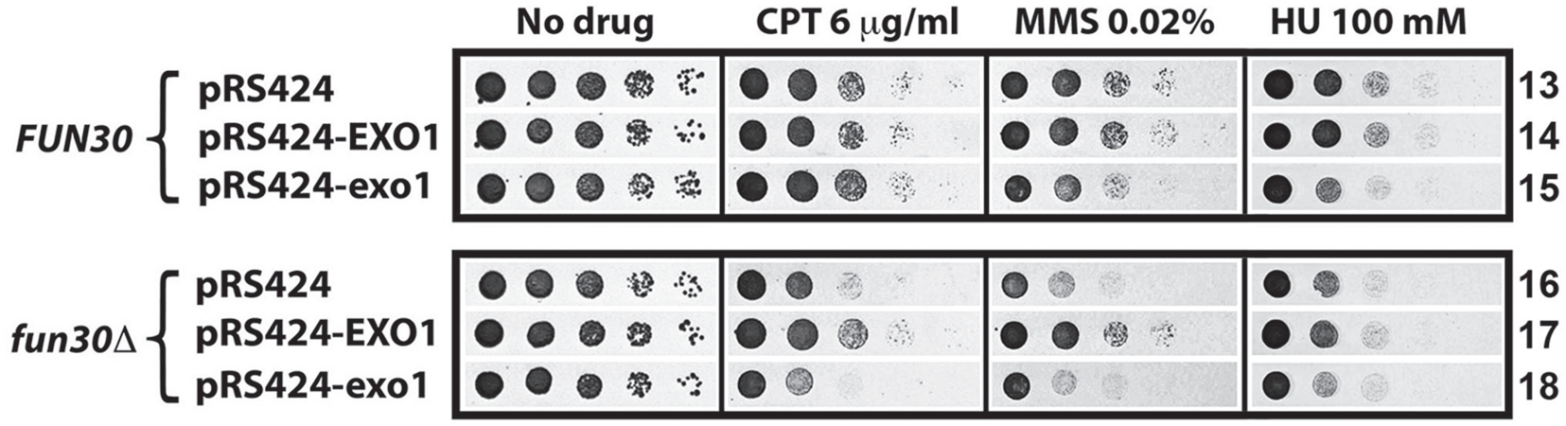
*EXO1* is a high copy suppressor of MMS sensitivity of *fun30Δ* mutant. Shown are growth phenotypes of strains 13–18 (S1 Table) on indicated media. Note pRS424 is a high copy 2μ vector, and pRS424-exo1 encodes the Exo1-D173A mutant.

Fun30 has been shown to play a direct role in the resection of DSB ends by both the Exo1- and Sgs1-dependent pathways, which is thought to underlie the contribution of Fun30 to CPT-resistance [23–25]. Consistently, overexpression of Exo1 suppresses CPT-sensitivity of *fun30Δ* cells [24] (Fig. 2, compare 16 and 17 on CPT). Here we examined if the function of Fun30 in MMS- and HU-resistance was also related to its role in DNA end resection. We showed that overexpression of Exo1, but not the nuclease deficient Exo1-D173A mutant, also suppressed MMS-sensitivity of *fun30Δ* mutant (Fig. 2, compare 17 and 18 with 16 on MMS). This result suggests that Fun30’s contribution to MMS-resistance is attributable to its function in DNA end resection. On the other hand, Exo1 overexpression did not seem to suppress HU-sensitivity of *fun30Δ* cells (Fig. 2).

### Fun30 deletion suppresses MMS and HU hypersensitivity caused by the lack of the Rad5/Mms2/Ubc13 dependent pathway of DNA damage tolerance

It is interesting that *fun30Δ* rendered *rad5-535* strains CCFY101 and ZGY005 more resistant to MMS (Fig. 1A). MMS-mediated base methylation halts the progression of DNA replication forks in S phase. Cells have evolved the error-free template switching and error-prone translesion synthesis pathways to bypass the damage, allowing DNA replication to proceed [7,8]. Rad5 is an E3 ubiquitin ligase involved in the template switching branch of DNA damage tolerance, and deletion of Rad5 results in a significant decrease in cellular resistance to MMS and HU [33]. Previous work showed that *rad5-535* had a weak DNA damage response phenotype compared to *rad5Δ* [32]. Consistently, we found that deletion of the *rad5-535* allele from ZGY005 sensitized the cells to MMS (S2 Fig., compare 31 with 7), and ectopically expressing *RAD5* in ZGY005 improved MMS-resistance (Fig. 1B, compare 11 with 9). These results, again, indicate that the Rad5-535 protein partially retains the function of Rad5 in DNA damage response.

**Fig 3:**
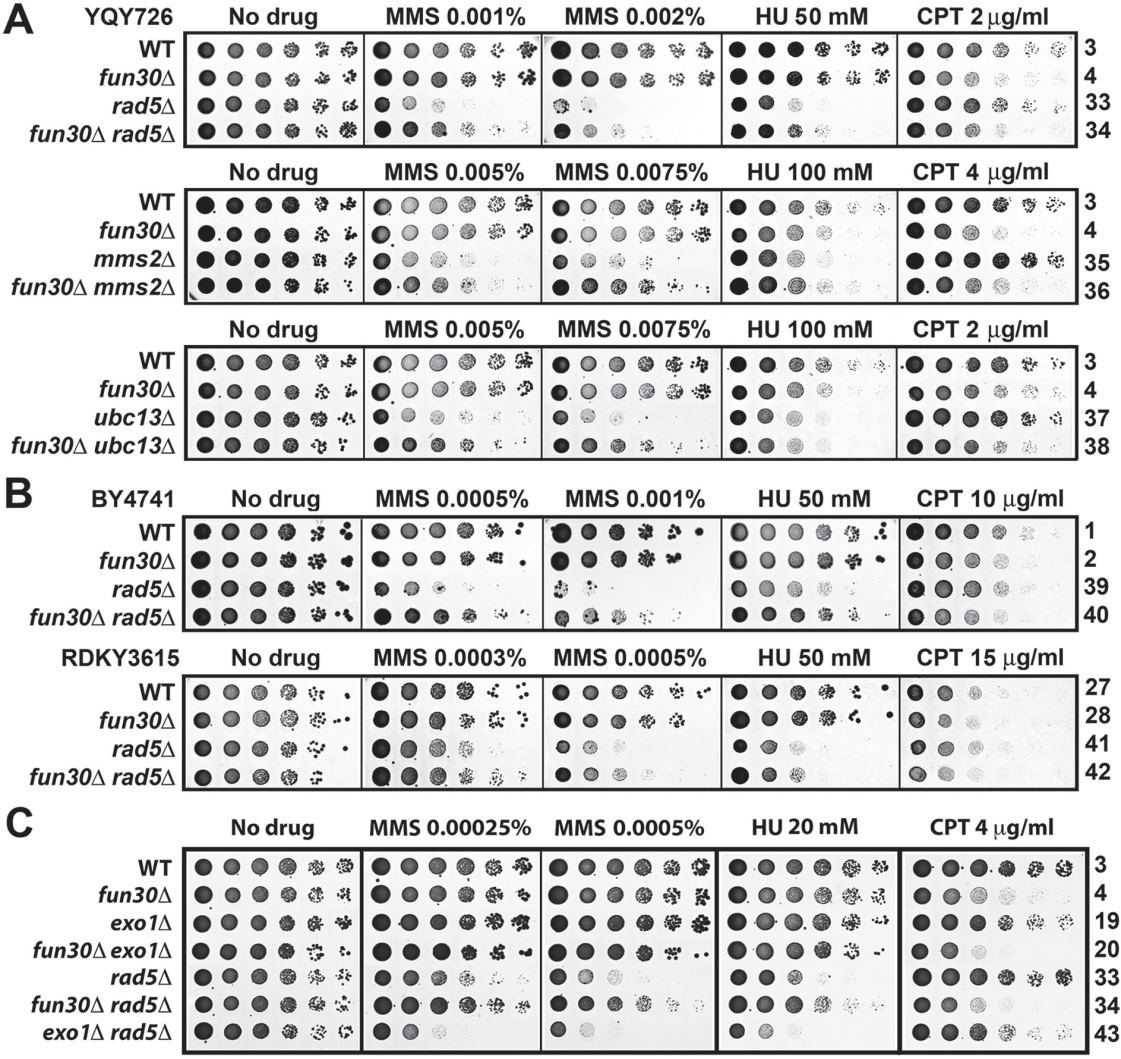
MMS- and HU-hypersensitivity of cells lacking Rad5/Ubc13/Mms2 is suppressed by *fun30Δ* but not *exo1Δ*. Shown are growth phenotypes of strains 3, 4, 19, 20, 27, 28, and 34 through 43 on indicated media.

The suppression of the defect of *rad5-535* mutant in MMS-resistance by *fun30Δ* prompted us to ask whether *fun30Δ* could also suppress the defect of *rad5Δ* mutant in MMS-tolerance. To address this question, we made a *rad5Δ fun30Δ* derivative of YQY726, and compared its tolerance to MMS with that of the *rad5Δ* and *fun30Δ* single mutants. As shown in Fig. 3A, *rad5Δ fun30Δ* double mutant was clearly more resistant to MMS than *rad5Δ* mutant. Moreover, *rad5Δ fun30Δ* mutant was also more resistant to HU than *rad5Δ* mutant (Fig. 3A). Therefore, *fun30Δ* suppresses the defect of *rad5Δ* in MMS- and HU-tolerance in the genetic background of YQY726. We also found this to be generally true in BY4741, RDKY3615 and ZGY005 backgrounds (Fig. 3B and S2 Fig.). On the other hand, *rad5Δ* had little effect on CPT-tolerance, and *fun30Δ* reduced CPT-tolerance of *rad5Δ* mutants (Fig. 3).

As an E3 ubiquitin ligase, Rad5 associates with the Mms2/Ubc13 E2 ubiquitin-conjugating enzyme to polyubiquitinate PCNA at lysine 164 (which has already been monoubiquitinated by Rad6/Rad18), which leads to DNA damage tolerance via the TS mechanism [33]. As such, Mms2 and Ubc13 function in the same pathway as Rad5. Consistently, *ubc13Δ* and *mms2Δ* reduced cellular tolerance to MMS and HU as did *rad5Δ* (Fig. 3A). We found *fun30Δ* to also suppress the defect of *ubc13Δ* and *mms2Δ* mutants in tolerance to MMS (Fig. 3A). *fun30Δ* moderately suppressed the defect of *mms2Δ* and *ubc13Δ* mutant in HU-tolerance (Fig. 3A, compare 35 and 36 on HU). Deletion of *mms2Δ* or *ubc13Δ* had little effect on CPT-tolerance, and Fun30 still contributed to CPT-tolerance in *mms2Δ* and *ubc13Δ* cells (Fig. 3A). Taken together, the above results indicate that Fun30 plays a negative role in MMS- and HU-tolerance, and a positive role in CPT-tolerance in cells lacking Rad5/Mms2/Ubc13.

### Deletion of Exo1 does not suppress the defect of *rad5Δ* mutant in MMS- and HU-resistance

Since Fun30 plays a role in DSB end resection, suppression of MMS- and HU-sensitivity of *rad5Δ* mutant by *fun30Δ* might be the result of a decline in the efficiency of DSB end resection. To test this hypothesis, we asked whether reducing DSB end resection by other means also suppresses MMS- and HU-sensitivity of *rad5Δ* mutant. It has been previously shown that *exo1Δ* reduced the efficiency of resection of a DSB to a similar extent as *fun30Δ* [23]. We showed here, however, that *exo1Δ* did not suppress *rad5Δ* sensitivity to MMS or HU (Fig. 3C, compare 43 with 33). Instead, *exo1Δ* rendered *rad5Δ* cells more sensitive to MMS and HU (Fig. 3C, compare 43 with 33). This result argues against the notion that *fun30Δ* makes *rad5Δ* cells more resistant to MMS and HU by reducing the efficiency of DSB end resection.

### Fun30 does not affect PCNA ubiquitination induced by MMS

**Fig 4:**
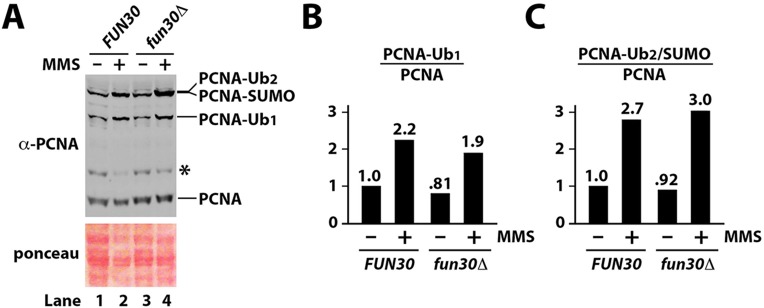
Fun30 does not affect MMS-induced PCNA modifications. (A). Exponentially growing BY4741 and BY4741 *fun30Δ* strains were treated with 0.02% MMS for 2 hours. Proteins in the cell extracts were analyzed by Western blot using an anti-PCNA antibody. Image of the ponceau-stained blot is also shown. The asterisk denotes a cross-reacting species. (B). The relative level of PCNA-Ub_1_ in each sample was measured as the ratio of the intensity of PCNA-Ub_1_ band over that of the PCNA band. PCNA-Ub_1_ level in *FUN30* cells without MMS treatment was taken as 1. (C). Quantification of PCNA-Ub_2_/SUMO relative to that of PCNA. PCNA-Ub_2_/SUMO level in *FUN30* cells without MMS treatment was taken as 1.

Given that Fun30 functionally interacts with Rad5/Ubc13/Mms2 (Fig. 3A), we asked whether Fun30 affected Rad5/Ubc13/Mms2-mdeaited PCNA polyubiquitination induced by replicative stress. PCNA from *FUN30* and *fun30Δ* cells before and after MMS treatment was analyzed by quantitative infrared western. As shown in Fig. 4, MMS induced a 2.2 fold increase in the level of PCNA monoubiquitination (PCNA-Ub_1_), and a 2.7 fold increase in the level of combined diubiquitination and sumoylation of PCNA (PCNA-Ub_2_/SUMO) in *FUN30* cells (compare lanes 1 and 2). In *fun30Δ* cells, MMS induced a 2.3 fold increase in PCNA-Ub_1_, and a 3.2 fold increase in PCNA- Ub_2_/SUMO (Fig. 4, compare lanes 3 and 4). Therefore, *fun30Δ* does not significantly affect PCNA-Ub_1_ or PCNA-Ub_2_/SUMO.

The co-migration of PCNA-Ub_2_ and PCNA-SUMO in our SDS-PAGE assay (Fig. 4A) prevented us from detecting PCNA-Ub_2_ specifically with an anti-PCNA antibody. To solve this problem, we pulled down His_6_-tagged PCNA and detected His_6_-PCNA-Ub_2_ with an anti-ubiquitin antibody. We found that *fun30Δ* did not significantly affect MMS-induced His_6_-PCNA-Ub_2_ (S3 Fig.). This result, together with data shown in Fig. 4, demonstrated that Fun30 does not affect the ubiquitination of PCNA induced by MMS.

### Suppression of MMS-sensitivity of *rad5Δ* cells by *fun30Δ* is dependent on Rad51

**Fig 5:**
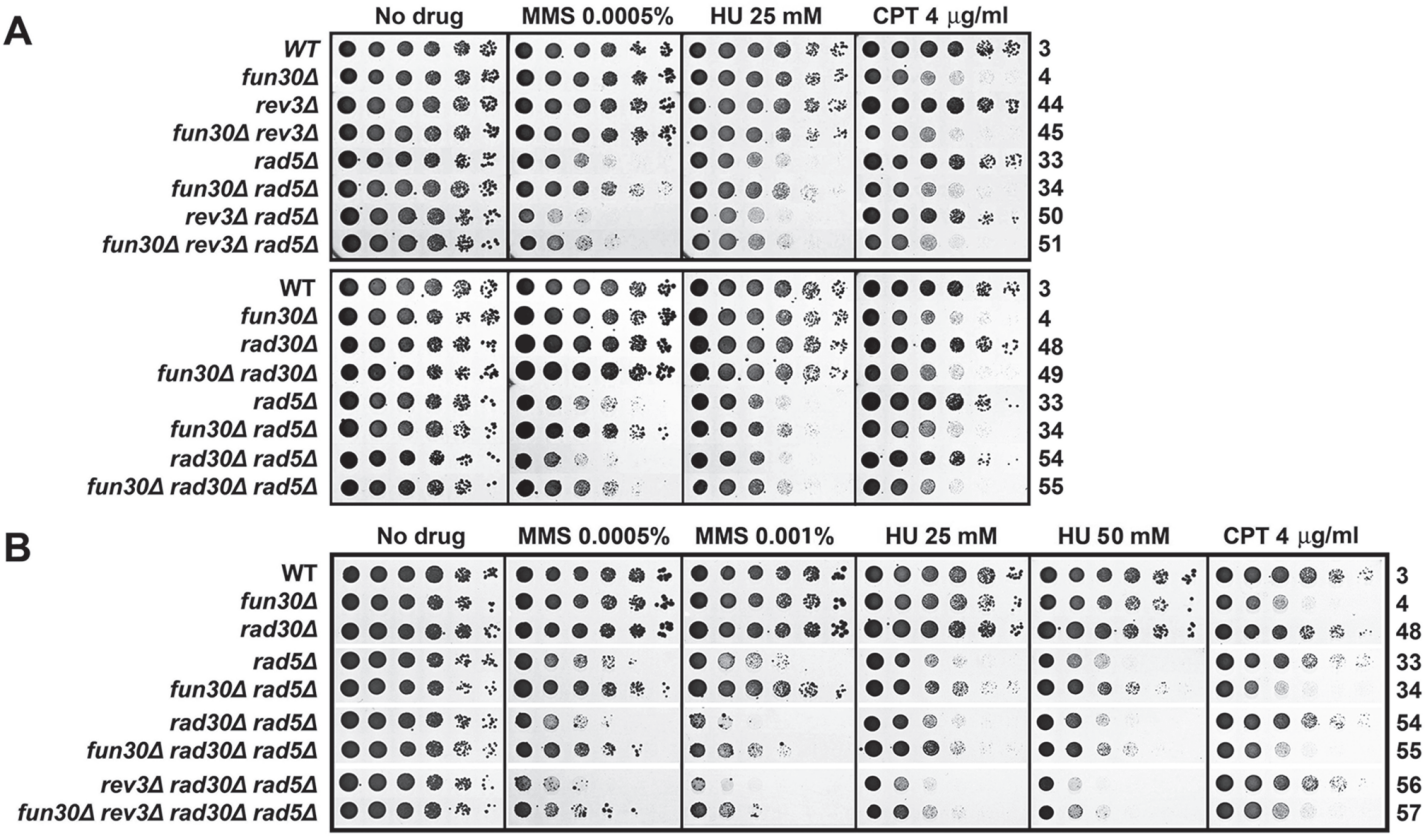
Fun30 can regulate cellular tolerance to genotoxins independently of TLS polymerases. Growth phenotypes of strains 3, 4, 33, 34, 44, 45, 48 through 51, and 54 through 57 on indicated media are shown.

It is possible that Fun30 plays a role in channeling MMS- or HU-induced DNA lesions to the Rad5-dependnet error-free damage bypass pathway, and these lesions are not bypassed effectively when Rad5 is absent. Along this line, deletion of Fun30 in a *rad5Δ* mutant may divert MMS- or HU-induced DNA lesions to an alternative repair/bypass mechanism(s), thereby promoting cellular tolerance to MMS. One possible alternative mechanism is the TLS pathway. If this were the case, then blocking TLS should prevent *fun30Δ* from suppressing *rad5Δ* sensitivity to MMS and HU. TLS is mediated by repair polymerase Polζ (Rev3/Rev7/Pol31) or Polη (Rad30) [34]. We found that deleting TLS component Rev3, Rev7 and Rad30 individually did not abolish the ability of *fun30Δ* to suppress MMS- and HU-sensitivity of *rad5Δ* mutant (Fig. 5A and S4 Fig.). We then deleted both *REV3* and *RAD30* from *rad5Δ* and *rad5Δ fun30Δ* mutants, and found that the *fun30Δ rev3Δ rad30Δ rad5Δ* quadruple mutant still exhibited an advantage over the *rev3Δ rad30Δ rad5Δ* triple mutant regarding resistance to MMS (Fig. 5B, compare 57 with 56). This result suggests that in the absence of both TLS polymerases, *fun30Δ* is still able to suppress *rad5Δ* sensitivity to MMS and HU.

**Fig 6:**
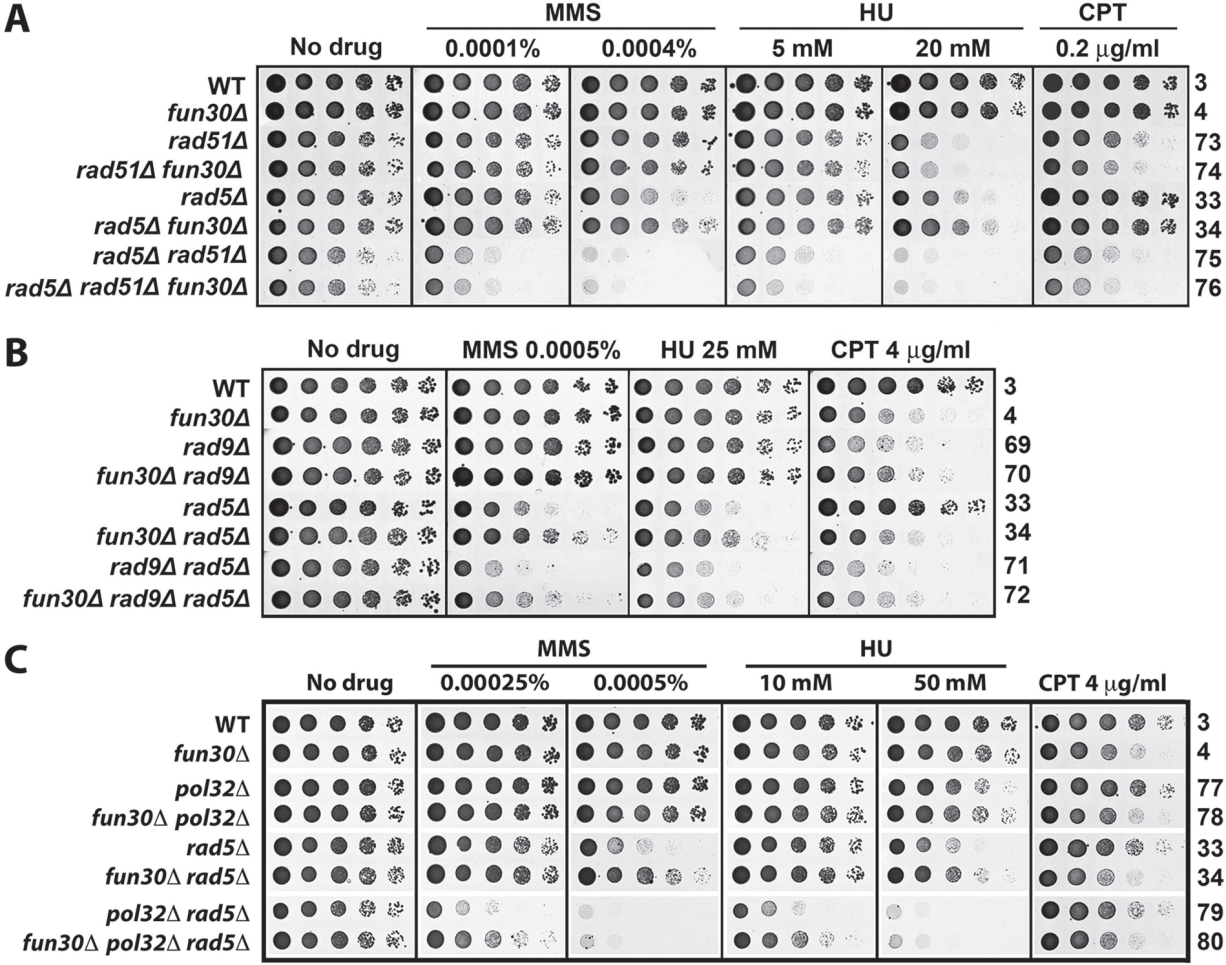
Suppression of MMS- and HU-hypersensitivity of *rad5Δ* mutant by *fun30Δ* is dependent on Rad51 but not Rad9 or Pol32. Shown are growth phenotypes of strains 3, 4, 33, 34, and 69 through 80 on indicated media.

We next examined if a DNA repair pathway or DNA damage checkpoint is involved in the suppression of *rad5Δ* by *fun30Δ* by testing whether *fun30Δ* could still improve MMS- and HU-tolerance of *rad5Δ* mutant lacking HR, BER, NER, NHEJ or DNA damage checkpoint. Specifically, we examined if suppression of *rad5Δ* defect by *fun30Δ* was dependent on Rad51 involved in HR, Mag1 in BER, Rad2 in NER, Yku70 in NHEJ, or Rad9 in DNA damage checkpoint. We found that *mag1Δ*, *rad2Δ*, *yku70Δ*, or *rad9Δ* did not abolish the ability of *fun30Δ* to suppress the defect in MMS- and HU-resistance of *rad5Δ* mutant (Fig. 6B and S5 Fig.). On the other hand, *fun30Δ* failed to suppress MMS- and HU-sensitivity of *rad5Δ* mutant when Rad51 was deleted (Fig. 6A). Therefore, the process that is involved in suppressing *rad5Δ* defect in MMS- and HU-tolerance by *fun30Δ* is a Rad51-dependent HR mechanism.

A Rad51-dependent process that is known to be inhibited by Fun30 is break-induced replication (BIR) [23,24,35,36]. Cells employ BIR to repair one-ended BSBs [37]. Stalled replication forks induced by genotoxins such as MMS and HU can potentially collapse/break, producing one-ended DSBs that could possibly be repaired by BIR. If this were the case, *fun30Δ* would derepress BIR allowing it to repair DSBs at replication forks that may accumulate in cells lacking the Rad5-dependent damage tolerance pathway. To test this hypothesis, we asked if *fun30Δ* suppression of *rad5Δ* sensitivity to MMS and HU is dependent on Pol32 that is required for BIR [38]. As shown in Fig. 6C, *fun30Δ* was still able to suppressed the sensitivity to MMS and HU of *rad5Δ* mutant deleted for Pol32 (compare 79 and 80 on 0.00025% MMS and 10 mM HU). Therefore, *fun30Δ-*induced suppression of *rad5Δ* sensitivity to MMS and HU can occur independently of Pol32 and, by inference, BIR.

### Fun30 and Srs2 function in separate pathways to regulate cellular resistance to genotoxin stress

**Fig 7:**
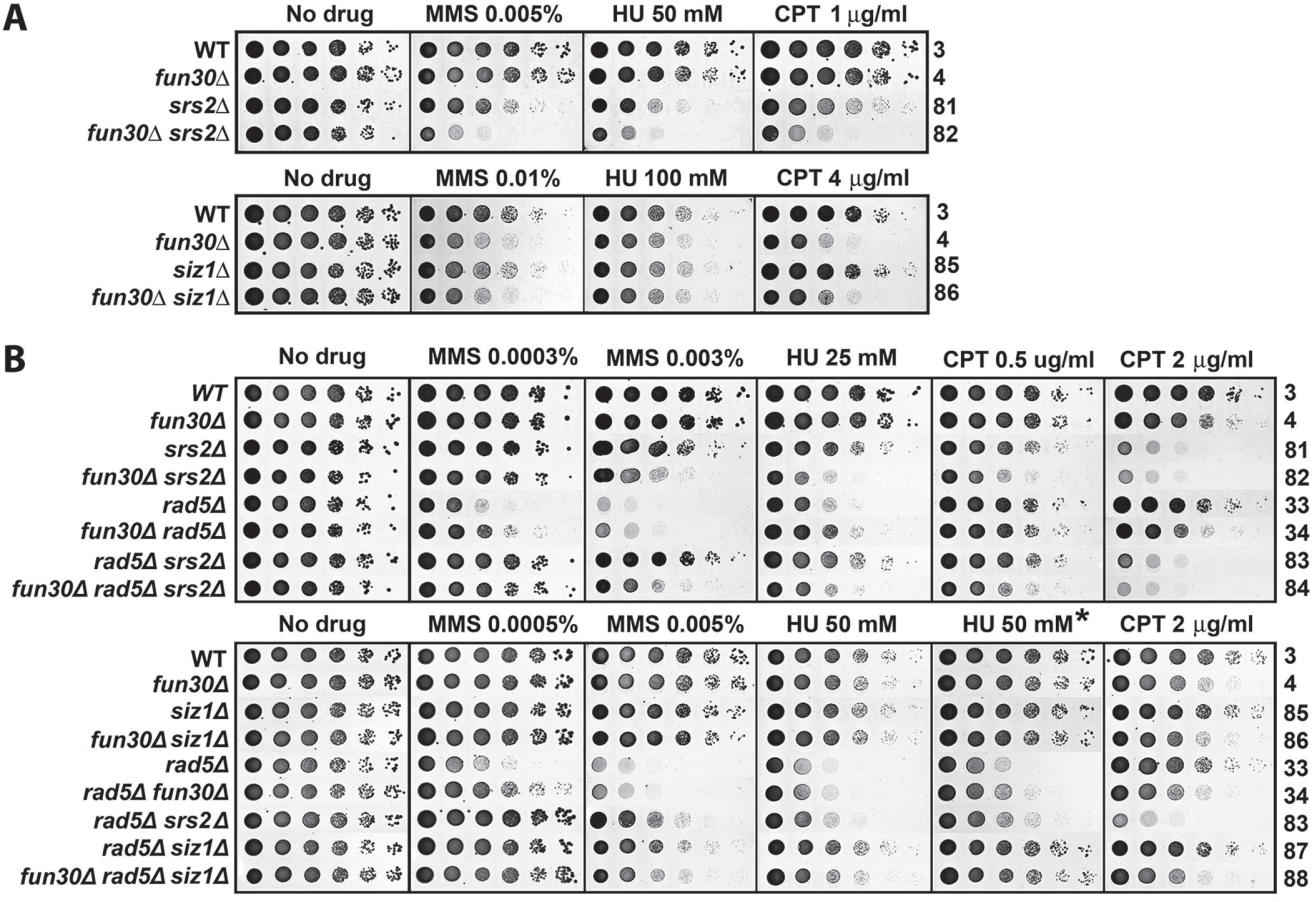
Genetic interactions among Fun30, Srs2 and Siz1 in genotoxin resistance. Shown are growth phenotypes of strains 3, 4, 33, 34, and 81 through 88 on indicated media. * Incubated for 4 days.

Srs2 has been shown to disrupt Rad51 nucleoprotein filaments and prevent undesirable DNA recombination during DNA replication that may generate toxic nucleoprotein intermediates [39,40]. In addition, Srs2 also contributes to DNA damage repair/tolerance and checkpoint recovery [41–43]. Similar to *fun30Δ*, *srs2Δ* reduces cellular tolerance to MMS under normal (*RAD5*) conditions, but suppresses MMS-sensitivity of *rad5Δ* mutant in a Rad51-dependent manner [44–46]. We found that *srs2Δ* made cells more sensitive to MMS, HU and CPT than *fun30Δ* (Fig. 7A, compare 81 with 4), indicating that Srs2 plays a greater role than Fun30 in cellular resistance to genotoxins. The *fun30Δ srs2Δ* double mutant was more sensitive to MMS, HU and CPT than the single mutants (Fig. 7A, compare 82 with 4 and 81), suggesting that Fun30 and Srs2 contribute to DNA damage response by independent mechanisms.

The role of Srs2 in preventing unwanted DNA recombination during DNA replication is linked to the Ubc9/Siz1-mediated sumoyltaion of PCNA in S phase (regardless of whether DNA damage is present) [45,46]. As Srs2 preferentially interacts with sumoylated PCNA, it is believed that sumoylated PCNA recruits Srs2 to DNA being replicated [45,46]. However, deletion of *SIZ1*, which abolishes PCNA sumoylation, results in a lesser effect on the tolerance to MMS, HU and CPT than *SRS2* deletion [45] (Fig. 7A, compare 85 and 81), suggesting that in addition to its Siz1-dependent function, Srs2 also functions in a Siz1-independent manner. We found that *siz1Δ* did not significantly exacerbate or mitigate the effect of *fun30Δ* on cellular tolerance to MMS, HU or CPT (Fig. 7A, compare 86 and 4). Therefore, Fun30’s function in cellular tolerance to genotoxic agents is not dependent on Siz1 (or PCNA sumoylation by inference). This also suggests that Fun30 function is separate from the Ubc9/Siz1-dependnet function of Srs2.

With respect to suppression of MMS- or HU-sensitivity of *rad5Δ* mutant, *srs2Δ* was more efficient than *fun30Δ* (Fig. 7B, top, compare 34 and 83) We noticed that deletion of Fun30 made *srs2Δ* and *siz1Δ* less effective in suppressing MMS- and HU-sensitivity of *rad5Δ* (Fig. 7B, bottom, compare 83 with 84, 87 with 88). These results suggest that Fun30 is required for efficient suppression of MMS or HU sensitivity of *rad5Δ* mutant by *srs2Δ* or *siz1Δ*.

### Fun30 delays the recovery of *rad5Δ* mutant from MMS induced G2/M checkpoint arrest

**Fig 8:**
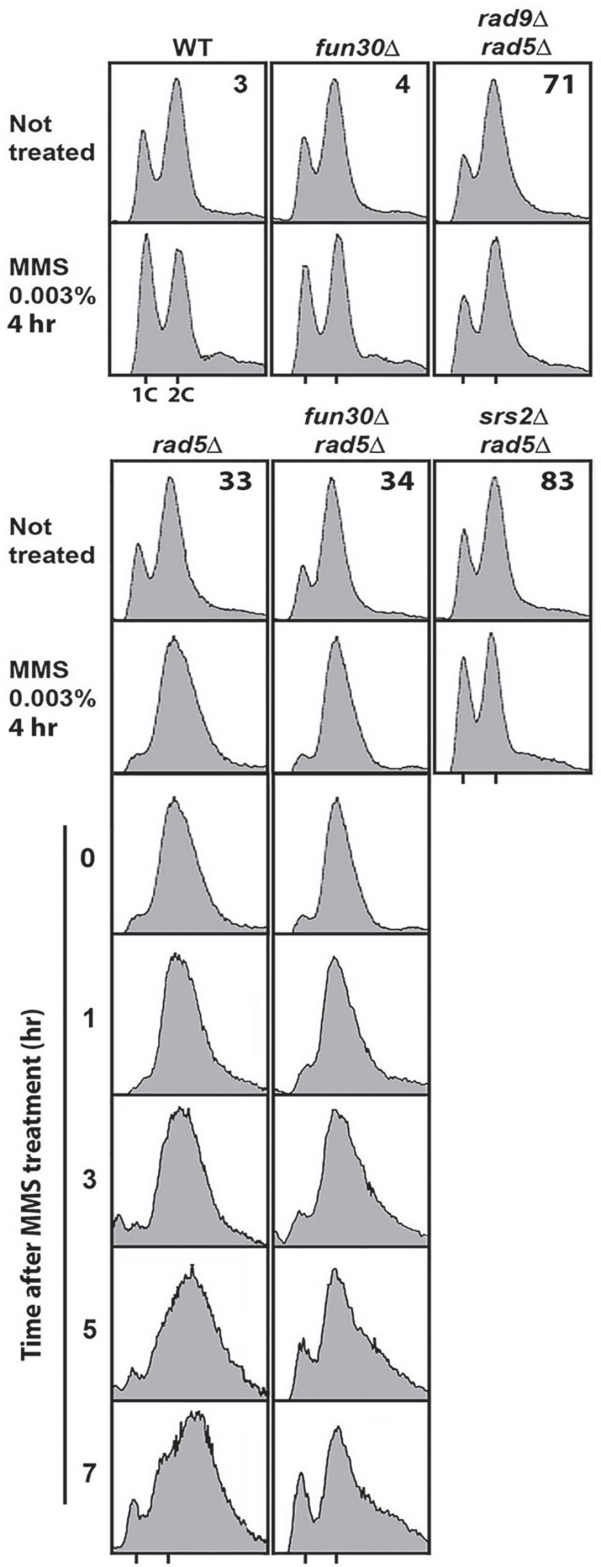
Fun30 delays the recovery of *rad5Δ* mutant from MMS induced G2/M arrest. Shown are FACS profiles of indicated strains with or without a 4 hr exposure to 0.003% MMS. FACS profiles of strains 33 and 34 at indicated time points after release from a 4 hr 0.003% MMS treatment are also shown.

It has been shown recently that the Rad5-dependent error-free DDT pathway is particularly important for tolerating chronic low dose UV (~ 0.2 J/m2) or MMS (~ 0.001%) treatment [47–49]. Chronic low dose UV or MMS treatment induces the accumulation of ssDNA gaps and a G2/M arrest in the absence of DDT [47–49]. Consistently, we showed here that treating *rad5Δ* cells with 0.003% MMS for 4 hr resulted in a G2/M arrest (Fig. 8, strain 33). The ssDNA gaps induced by UV or MMS treatment in DDT defective cells are subject to HR repair [47–49]. In line with this notion, *srs2Δ* suppresses the sensitivity of DDT-deficient cells to low dose UV or MMS, and prevents G2/M arrest [47,48] (Fig. 8, compare 83 with 33). Given that *fun30Δ* partially suppressed the defect of *rad5Δ* sensitivity to MMS (Fig. 3A), we wondered if *fun30Δ* also prevented G2/M-arrest of *rad5Δ* mutant treated with MMS. We found that *rad5Δ fun30Δ* double mutant was still arrested in G2/M despite that it is more resistant to MMS than *rad5Δ* mutant (Fig. 8, compare 34 with 33). Therefore, it seems a G2/M arrest is not always correlated with the same degree of MMS-sensitivity. In line with this notion, we found that although *rad9Δ rad5Δ* double mutant was more sensitive to MMS than *rad5Δ* mutant (Fig. 6B, compare 71 with 33), it was not arrested in G2/M by MMS (Fig. 8, compare 71 with 33).

MMS or UV induced G2/M arrest of DDT defective cells is gradually reversed after release from the treatment due to the repair of ssDNA gaps by HR [47–49]. We found that *fun30Δ* accelerated the recovery of *rad5Δ* mutant from MMS induced G2/M arrest (Fig. 8, compare 34 with 33). It is worth noting that the FACS profile of *rad5Δ* cells appeared distorted during the reversal of MMS induced G2/M arrest (Fig. 8, strain 33). This may reflect a dysregulation of DNA synthesis and/or cell division. This phenomenon was partially suppressed by *fun30Δ* (Fig. 8, compare 34 with 33). These results, together with our finding that suppression of *rad5Δ* by *fun30Δ* was Rad51-dependent (Fig. 6A), suggest that Fun30 negatively regulates HR-mediated repair of MMS-induced DNA damage in *rad5Δ* cells.

### Role of the CUE domain of Fun30 in regulating cellular tolerance to genotoxins

**Fig 9:**
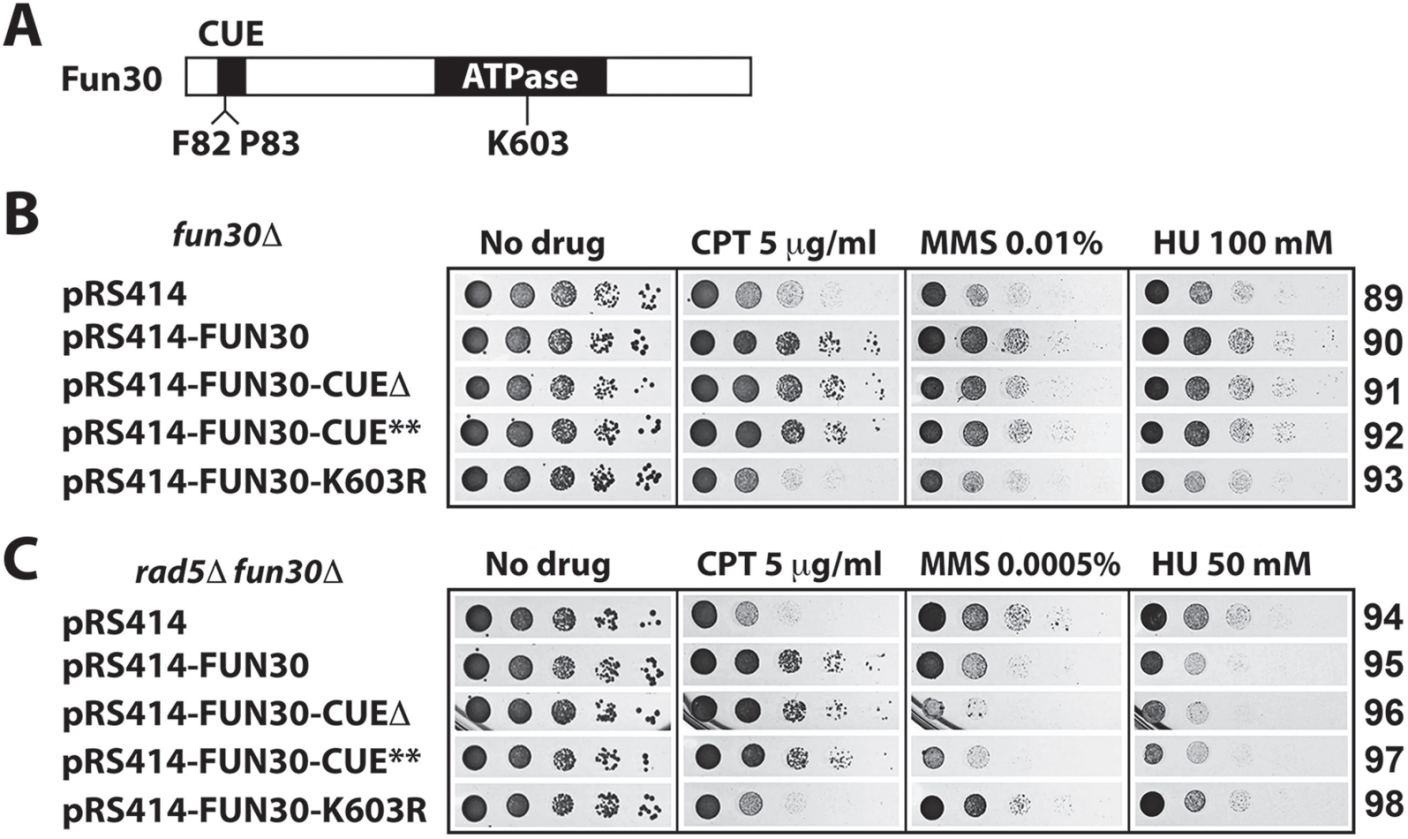
Analysis of the role of the Fun30 CUE motif in genotoxin resistance. (A) Schematic of Fun30 protein. The CUE motif and ATPase domain are indicated. (B) The CUE motif of Fun30 is not required for genotoxin resistance. Growth phenotypes of strains 89 through 93 on indicated media are shown. Note pRS414-FUN30-CUE** encodes a Fun30 mutant with F82 and P83 in the CUE motif replaced by alanines. (C) The CUE motif restricts Fun30’s ability to negatively regulate MMS-tolerance. Shown are growth phenotypes of strains 94 through 98 on indicated media.

Fun30 has a CUE (Coupling of Ubiquitin conjugation to ER degradation) motif near its N-terminus [22] (Fig. 9A). This is a putative ubiquitin-binding motif composed of ~35 amino acids in which a conserved FP (phenylalanine-proline) motif is potentially important for ubiquitin binding [50]. The CUE motif of Fun30 has been shown to be dispensable for the role of Fun30 in DSB end resection [23]. To examine whether CUE is required for Fun30 function in genotoxin-resistance, we introduced plasmids encoding intact Fun30, Fun30-CUEΔ (CUE deleted), Fun30-CUE** (conserved FP in CUE replaced with alanines), as well as Fun30-K603R (ATPase deficient, used as a negative control) into *fun30Δ* mutant. As shown in Fig. 9B, ectopic expression of Fun30 restores CPT-, MMS- and HU-resistance to *fun30Δ* mutant (compare 90 with 89), which was dependent on the ATPase/chromatin remodeling activity of Fun30 (compare 93 with 89). We found that mutating the CUE domain did not significantly affect the ability of Fun30 to promote resistance to CPT, MMS and HU (Fig. 9B, compare 91 and 92 with 90).

We next asked if the CUE domain was required for Fun30 mediated negative regulation of MMS- and HU-resistance in *rad5Δ* mutant. We showed that ectopic expression of Fun30 made *rad5Δ fun30Δ* mutant more sensitive to MMS and HU (Fig. 9C, compare 95 with 94), confirming the inhibitory effect of Fun30 on MMS- and HU-resistance. Interestingly, expressing Fun30-CUEΔ or Fun30-CUE** reduced MMS-resistance to a greater extent than expressing wild type Fun30 (Fig. 9C, compare 96 and 97 with 94 and 95 on MMS). Disrupting CUE domain had a similar, albeit lesser, effect on HU-resistance compared to MMS-resistance (Fig. 9C, compare 96 and 97 with 94 on HU). These results suggest that the CUE domain serves to restrict the function of Fun30 in negatively regulating MMS- and perhaps also HU-tolerance of *rad5*Δ cells.

## Discussion

We found that the Fun30 chromatin remodeler contributes to cellular resistance to MMS and HU in addition to its previously demonstrated function in CPT resistance. Therefore, Fun30 likely plays a role in the repair of DNA damages induced by genotoxins in general. On the other hand, interestingly, deletion of Fun30 differentially affects MMS/HU resistance and CPT resistance when the error-free DNA damage tolerance pathway is compromised. Fun30 is still required for efficient resistance to CPT, but has an inhibitory effect on tolerance to MMS and HU in cells lacking Rad5/Mms2/Ubc13 mediated DDT mechanism. Therefore, Fun30 may positively or negatively modulate cellular response to genotoxic stress depending on the genetic context of the cell.

### Fun30 promotes cellular resistance to genotoxins MMS and HU

We showed in this work that Fun30 contributes to cellular resistance to MMS and HU in addition to CPT (Fig. 1). Recent studies have established a direct role of Fun30 in facilitating long-range DSB end resection [23–25]. This, together with the finding that overexpression of Exo1 can compensate for the loss of Fun30 function in CPT resistance, indicates that Fun30 helps to repair CPT-induced DSBs by aiding in DSB end resection needed for HR. The fact Exo1 overexpression also suppresses MMS sensitivity of *fun30Δ* mutant (Fig. 2) points to a role of DNA end resection in the repair of MMS induced DNA damage. However, unlike CPT that induce DSBs by trapping topoisomerase covalently linked to nicked DNA, MMS methylates nucleotides thereby stalling DNA replication, which results in the uncoupling of DNA helicase and polymerase activities at the replication fork and formation of single stranded gaps [11–13]. Arrested replications forks are subject to repair by the damage tolerance pathways, and if left unrepaired, could potentially collapse and yield DSBs. Therefore, MMS induces ssDNA gaps and potentially also DSBs. There has been evidence suggesting a role for Exo1 in processing MMS-induced ssDNA gaps [51]. We envision that Fun30 may promote MMS-resistance by aiding in the processing of ssDNA gaps and/or DSBs at arrested replication forks.

Overexpression of Exo1 does not seem to suppress HU sensitivity of *fun30Δ* mutant (Fig. 2). Therefore, improving DNA resection in *fun30Δ* cells is not sufficient to restore HU-resistance. This may be because HU does not damage/modify nucleotides as MMS does, and is less efficient in blocking DNA replication and generating ssDNA gaps and/or DSBs. Alternatively, or in addition, Fun30 may have another function besides DNA end resection that is important for HU-resistance.

### Fun30 negatively regulates cellular tolerance to MMS and HU in the absence of Rad5-dependent error-free DNA damage tolerance pathway

Replicative stress poses a serious threat to genome integrity as it may result in ssDNA gaps at stalled replication forks and potentially also DSBs if the stalled replication forks collapse [11–13]. The Rad5/Mms2/Ubc13 dependent DNA damage tolerance pathway plays an important role in the restart of arrested replication forks, and its absence renders cells hypersensitive to MMS and HU [33]. We found that *fun30Δ* partially restores the resistance of *rad5Δ*, *mms2Δ* or *ubc13Δ* mutant to MMS and HU (Fig. 3A and 3B). This is unlikely the result of *fun30Δ*-induced decline in DNA end resection, as *exo1Δ* which also causes a reduction in DNA end resection does not suppress *rad5Δ* sensitivity to MMS and HU (Fig. 3C). We envision that Fun30 is inhibitory to a mechanism other than DNA end resection that helps repair or bypass DNA damages induced by MMS or HU in cells lacking Rad5/Mms2/Ubc13. We presented evidence indicating that the putative mechanism inhibited by Fun30 is unlikely to be NER, BER, NHEJ, TLS or Rad9-dependent DNA damage checkpoint (Fig. 5, Fig. 6, S4 Fig. and S5 Fig.). On the other hand, such a mechanism is dependent on Rad51 (Fig. 6A), and is thus an HR mediated process. It has been well documented that the Srs2 helicase serves to prevent undesirable DNA recombination during DNA replication that could lead to harmful nucleoprotein intermediates [39,40]. We found evidence that Fun30 and Srs2 function in separate pathways to regulate genotoxin resistance (Fig. 7).

The Rad5-dependent error-free DDT pathway has been shown recently to be uniquely important for cellular tolerance to low dose UV or MMS treatment [47–49]. In the absence of this pathway, cells accumulate of ssDNA gaps, which triggers a G2/M arrest. Upon release from the arrest, cells gradually reenter the cell cycle [47]. This recovery is believed to be promoted by HR-mediated repair of ssDNA gaps [47]. We showed in this report that Fun30 plays an inhibitory role in the recovery of *rad5Δ* cells from MMS-induced G2/M arrest (Fig. 8). This result suggests that Fun30 negatively regulates HR-mediated repair of ssDNA gaps induced by MMS.

Fun30 is a founding member of a subfamily of chromatin remodelers that share a characteristic CUE motif [22]. This putative ubiquitin-binding motif has been shown to be dispensable for Fun30’s role in DSB end resection [23]. We showed that CUE is also not required for cellular resistance to CPT, MMS and HU (Fig. 9B). Interestingly, mutating the CUE domain of Fun30 made *rad5Δ* cells more sensitive to MMS (Fig. 9C), suggesting that the CUE motif acts to restrict the ability of Fun30 to inhibit a putative mechanism for countering MMS induced replicative stress. It will be interesting to investigate if CUE regulates Fun30 function by binding to ubiquitin on another part of Fun30 or a separate regulator.

## Conclusions

In summary, we think Fun30 performs two different functions in cellular response to DNA damage. One is the promotion of DSB repair by aiding in DSB end resection. In this process, Fun30 is recruited to DSBs and is believed to help overcoming the chromatin barrier to DSB end resection [23–25]. The other is the negative regulation of cellular tolerance to replicative stress caused by DNA damage, which is not attributable to Fun30’s role in DSB end resection. As a chromatin remodeler, Fun30 has been shown to promote the repression of 223 genes in the genome and the formation and maintenance of heterochromatin in yeast [22,52,53]. We envision that Fun30 performs these functions by making chromatin structure more stable (or less dynamic), thereby rendering DNA in chromatin more refractory to DNA transactions including homologous recombination (HR). Along this line, we propose that Fun30 inhibits an HR-mediated DDT pathway, and derepression of this HR-dependent pathway by *fun30Δ* suppresses MMS-sensitivity caused by the loss of Rad5-dependent DDT pathway.

## Supporting Information

S1 Fig CCFY101 and ZGY005 strains bear the *rad5-535* allele in them whereas BY4741 and YQY726 have the *RAD5* allele. Whether the *rad5-535* mutation is present in a strain can be examined by digesting the 336 bp 1476–1812 bp fragment of the *RAD5* ORF with MnlI. (A) There is a single MnlI site in this fragment at coordinate 1657. Digestion of the wild type *RAD5* fragment with MnlI yields a 182 bp and a 155 bp fragment. (B) The *rad5-535* mutation (G1603A) results in an extra MnlI site at 1595. Digestion of the *rad5-535* fragment with MnlI produces three fragments (120 bp, 62 bp and 155 bp). An incomplete digestion (cutting at only one of the two MnlI sites) generates a 217 bp and a 182 bp fragment. (C) *RAD5* alleles in strains CCFY101, ZGY005, BY4741, YQY726 were analyzed. The 1476–1812 bp fragment of the *RAD5* ORF from each strain was PCR amplified and digested with MnlI. Gel electrophoresis of the digestion products revealed that the digestions were not complete. The result indicates that CCFY101 and ZGY005 strains bear the *rad5-535* allele whereas BY4741 and YQY726 the *RAD5* allele.
(TIF)

S2 Fig The Rad5-535 protein partially retains the function of Rad5 in DNA damage tolerance. Growth phenotypes of strains 7, 8, 31 and 32 on indicated media are shown.
(TIF)

S3 Fig Fun30 does not affect MMS-induced PCNA polyubiquitination. Exponentially growing strains RDKY6649 and YXB013-106 were treated with 0.02% MMS for 2 hours. Cells from 100 ml culture were harvested, washed, and lysed by treatmnet with a lysis buffer containing 1.2 M NaOH and 4.6% β-mercaptoethanol. Proteins in the lysate were precipiated by treament with 25% trichloroacetic acid, washed with acetone, and resuspended in a buffer containing 6M guanidine hydrochloride, 0.1 M Na_2_HPO_4_, and 10 mM imidazole. Nickel-nitrilotriacetic acid (Ni-NTA)-agarose affinity pull-down of His-tagged PCNA was performed according to the manufacturer's instruction (Qiagen). Briefly, the proteins were miexed with 100 μl of Ni-NTA resin and incubated for 2 hours at room temperature. The resin was washed with wash beffer 1 (1.5 M guanidine hydrochloride, 25 mM Na_2_HPO_4_, and 18 mM imidazole, 18 mM TrisCl, pH 6.8) and then wash buffer 2 (20 mM imidazole, 25 mM TrisCl, pH 6.8). Proteins were eluted by boiling for 5 minutes in Laemmli buffer. Protein samples were analyzed by SDS-PAGE and Western blot. The blot was then separately probed with anti-ubiquitin (α-HA) and α -PCNA antibodies. The asterisk denotes a cross-reacting species.
(TIF)

S4 Fig Fun30’s function in cellular resistance to genotoxins is independent on Rev7. Growth phenotypes of strains 3, 4, 33, 34, 46, 47, 52 and 53 on indicated media are shown.
(TIF)

S5 Fig Suppression of MMS- and HU-hypersensitivity of *rad5Δ* mutant by *fun30Δ* is independent on Mag1, Rad2 or Yku70. Shown are growth phenotypes of strains 3, 4, 33, 34, and 58 through 68 on indicated media.
(TIF)

S1 Table Yeast strains. (PDF)
