# Supplementary figures and images for: Functions of Fun30 Chromatin Remodeler in Regulating Cellular Resistance to Genotoxic Stress
Source: PLoS One. 2015 Mar 25;10(3):e0121341. doi: 10.1371/journal.pone.0121341 (PMC4373758; doi:10.1371/journal.pone.0121341)

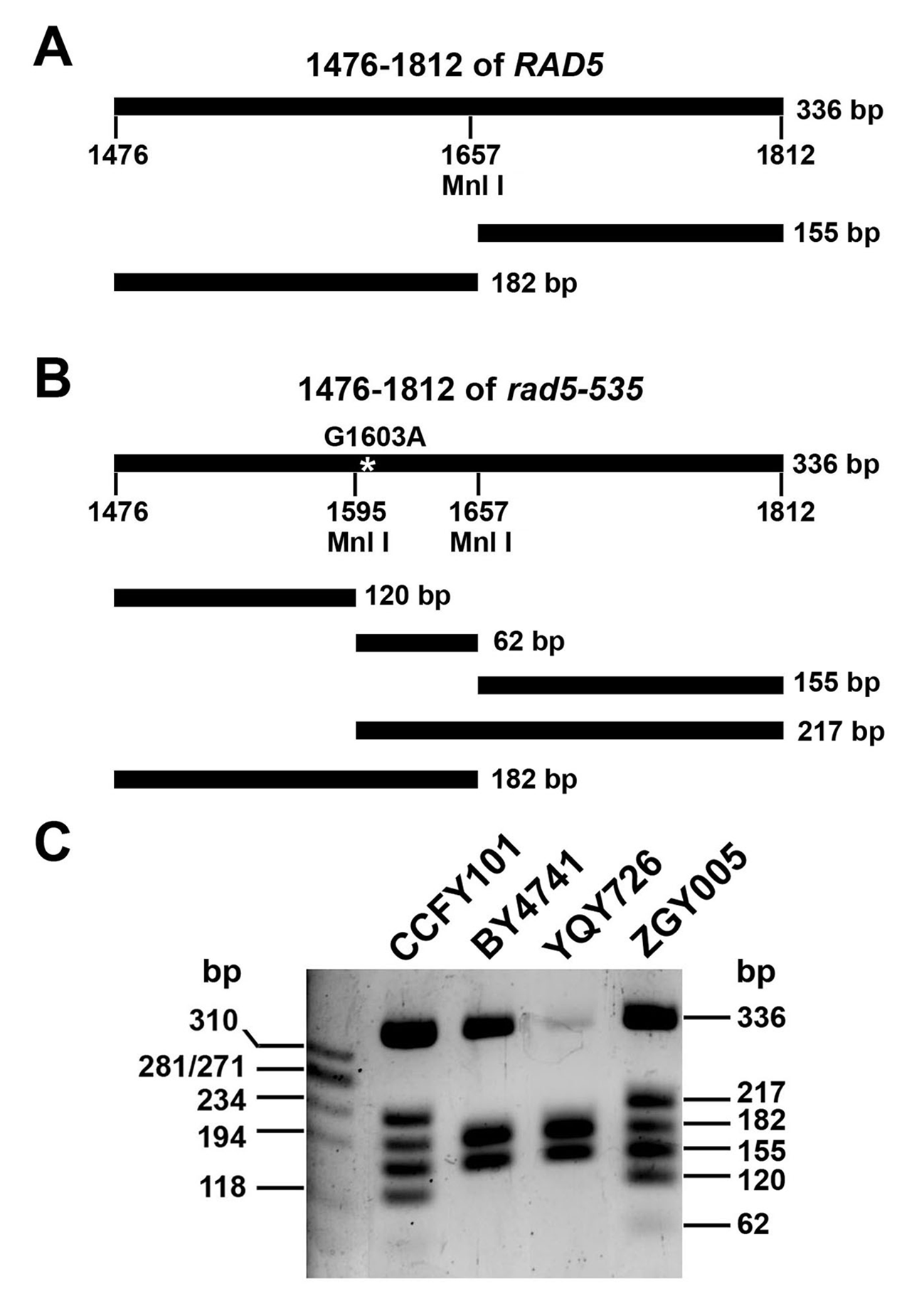

Supplement: S1 Fig — Whether the rad5-535 mutation is present in a strain can be examined by digesting the 336 bp 1476–1812 bp fragment of the RAD5 ORF with MnlI. (A) There is a single MnlI site in this fragment at coordinate 1657. Digestion of the wild type RAD5 fragment with MnlI yields a 182 bp and a 155 bp fragment. (B) The rad5-535 mutation (G1603A) results in an extra MnlI site at 1595. Digestion of the rad5-535 fragment with MnlI produces three fragments (120 bp, 62 bp and 155 bp). An incomplete digestion (cutting at only one of the two MnlI sites) generates a 217 bp and a 182 bp fragment. (C) RAD5 alleles in strains CCFY101, ZGY005, BY4741, YQY726 were analyzed. The 1476–1812 bp fragment of the RAD5 ORF from each strain was PCR amplified and digested with MnlI. Gel electrophoresis of the digestion products revealed that the digestions were not complete. The result indicates that CCFY101 and ZGY005 strains bear the rad5-535 allele whereas BY4741 and YQY726 the RAD5 allele. (TIF) [file pone.0121341.s001.tif]

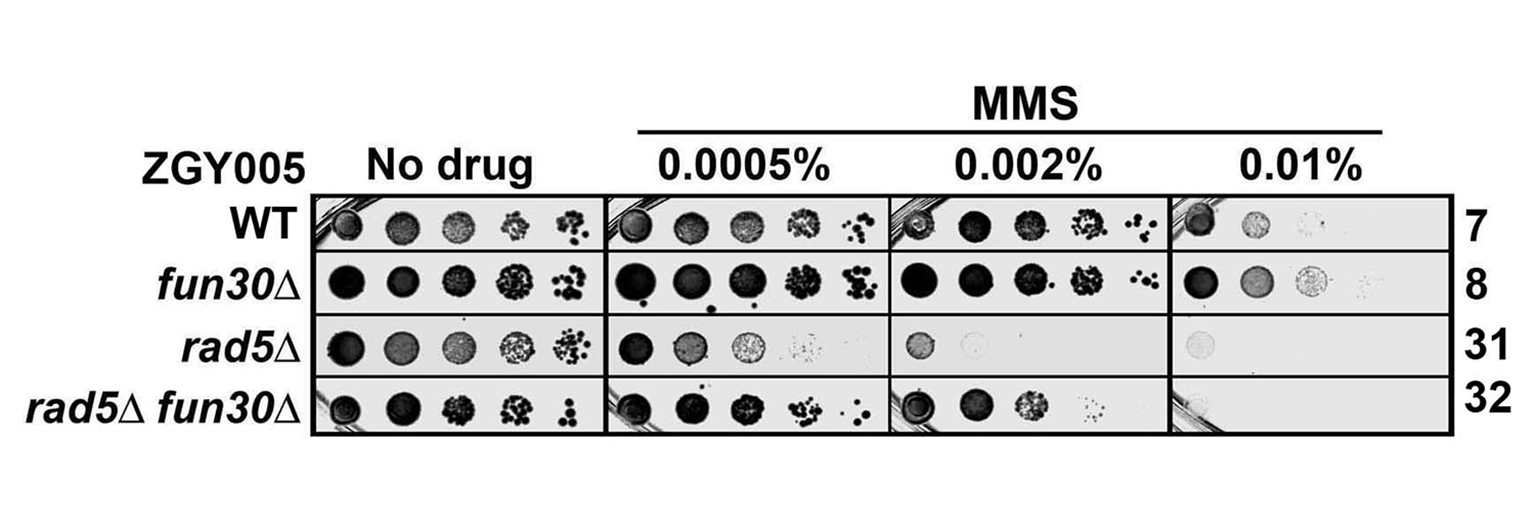

Supplement: S2 Fig — Growth phenotypes of strains 7, 8, 31 and 32 on indicated media are shown. (TIF) [file pone.0121341.s002.tif]

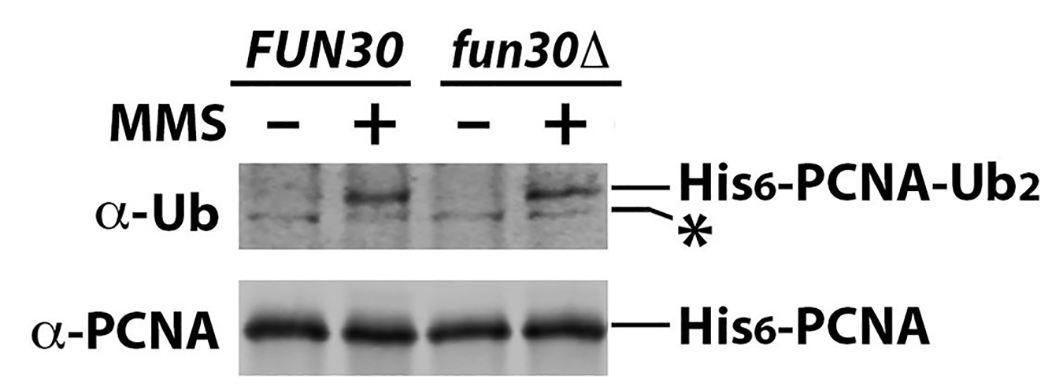

Supplement: S3 Fig — Exponentially growing strains RDKY6649 and YXB013-106 were treated with 0.02% MMS for 2 hours. Cells from 100 ml culture were harvested, washed, and lysed by treatmnet with a lysis buffer containing 1.2 M NaOH and 4.6% β-mercaptoethanol. Proteins in the lysate were precipiated by treament with 25% trichloroacetic acid, washed with acetone, and resuspended in a buffer containing 6M guanidine hydrochloride, 0.1 M Na2HPO4, and 10 mM imidazole. Nickel-nitrilotriacetic acid (Ni-NTA)-agarose affinity pull-down of His-tagged PCNA was performed according to the manufacturer's instruction (Qiagen). Briefly, the proteins were miexed with 100 μl of Ni-NTA resin and incubated for 2 hours at room temperature. The resin was washed with wash beffer 1 (1.5 M guanidine hydrochloride, 25 mM Na2HPO4, and 18 mM imidazole, 18 mM TrisCl, pH 6.8) and then wash buffer 2 (20 mM imidazole, 25 mM TrisCl, pH 6.8). Proteins were eluted by boiling for 5 minutes in Laemmli buffer. Protein samples were analyzed by SDS-PAGE and Western blot. The blot was then separately probed with anti-ubiquitin (α-HA) and α -PCNA antibodies. The asterisk denotes a cross-reacting species. (TIF) [file pone.0121341.s003.tif]

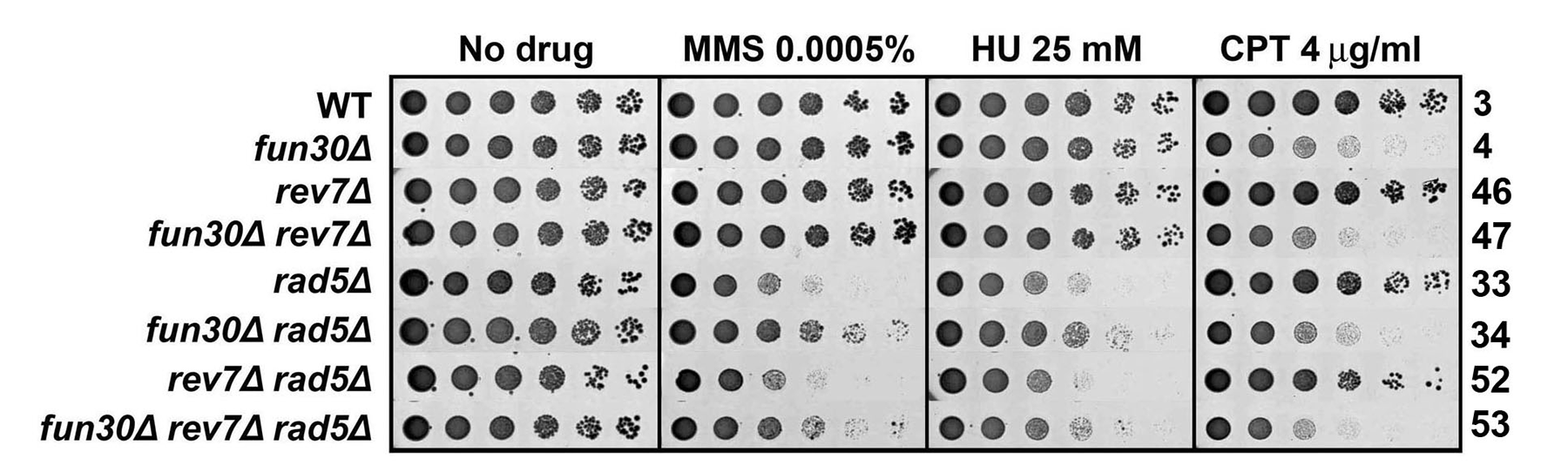

Supplement: S4 Fig — Growth phenotypes of strains 3, 4, 33, 34, 46, 47, 52 and 53 on indicated media are shown. (TIF) [file pone.0121341.s004.tif]

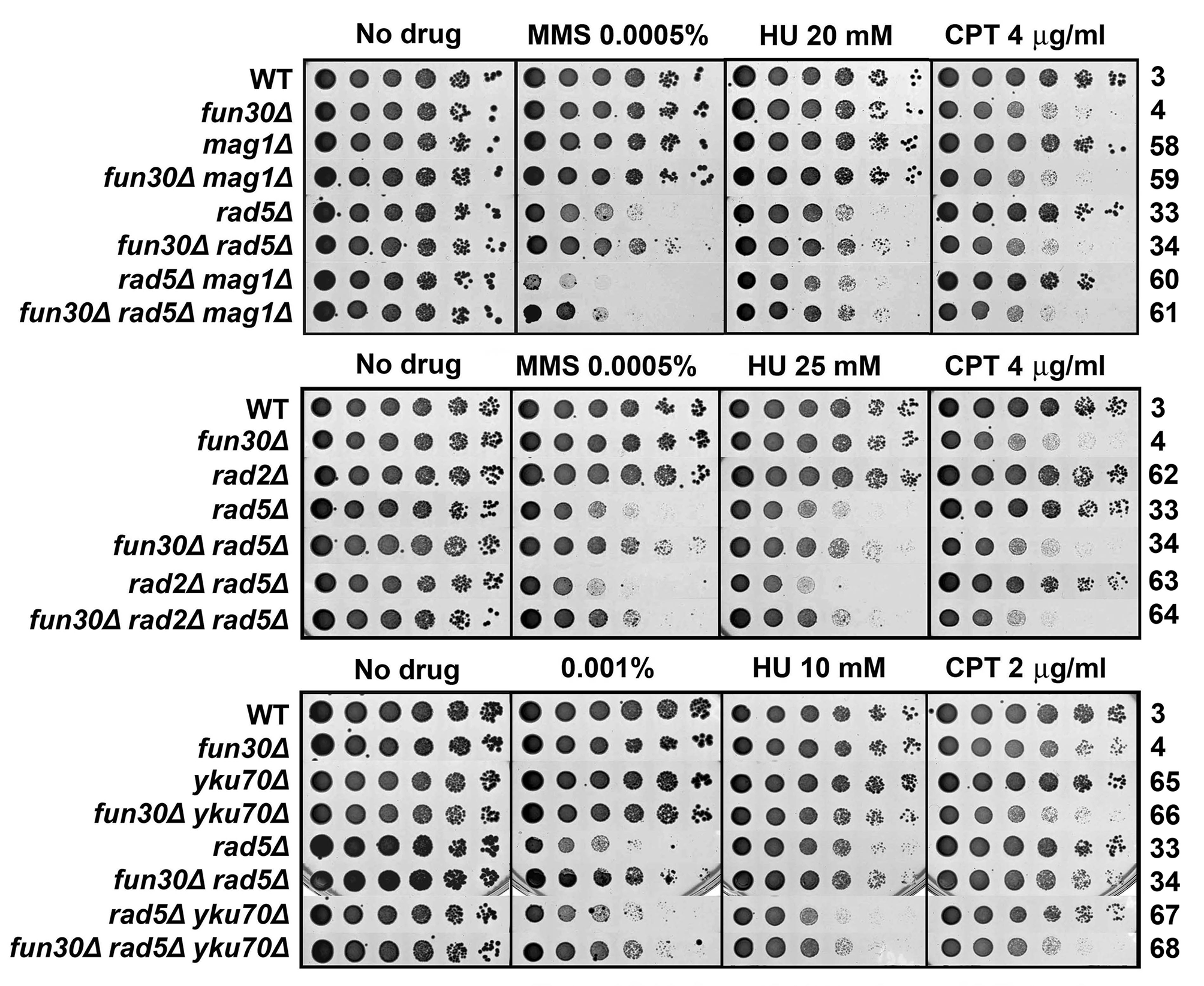

Supplement: S5 Fig — Shown are growth phenotypes of strains 3, 4, 33, 34, and 58 through 68 on indicated media. (TIF) [file pone.0121341.s005.tif]
