# Supplementary material for: Functions of Fun30 Chromatin Remodeler in Regulating Cellular Resistance to Genotoxic Stress
Source: PLoS One. 2015 Mar 25;10(3):e0121341. doi: 10.1371/journal.pone.0121341 (PMC4373758; doi:10.1371/journal.pone.0121341)
Supplement: S1 Table — (PDF) [file pone.0121341.s006.pdf]

S1 Table Yeast strains

Number	Name	Genotype	Source
1	BY4741	*MATa his3Δ1 leu2Δ0 met15Δ0 ura3Δ0*	Open Biosystems
2	YXB013-1	BY4741, *fun30Δ::KanMX*	Open Biosystems
3	YQY726	*MATa ura3 his3 ade2 can1 trp1 his5 LEU2-GAL10-FLP hhf1Δ::HIS3 hhf2Δ::LEU2 hht1Δ::LoxP FRT-hml::URA3* [cir^{o}] + pRS412-*HHT2-HHF2*	This work
4	YXB013-2	YQY726, *fun30Δ::NatMX*	This work
5	CCFY101	*MATα ade2-1 can1-100 his3-11,15 leu2–3,112 trp1-289 ura3-1 hmrΔe::TRP1 Tel VR-URA3 RDN1::ADE2-CAN1*	Ref. 1
6	YXB013-3	CCFY101, *fun30Δ::NatMX*	This work
7	ZGY005	*MATa leu2-3,112 ura3-1 his3-11,15 trp1-ade2-1 can1-100 hmr::ADE2 pol30Δ::hisG* + pBL211(*POL30-URA3*)	Ref. 2
8	YXB013-4	ZGY005, *fun30Δ::NatMX*	This work
9	YXB013-5	CCFY101 + pRS415	This work
10	YXB013-6	YXB013-3 + pRS415	This work
11	YXB013-7	CCFY101 + pRAD5	This work
12	YXB013-8	YXB013-3 + pRAD5	This work
13	YXB013-81	YQY726 + pRS424	This work
14	YXB013-82	YQY726 + pRS424-EXO1	This work
15	YXB013-83	YQY726 + pRS424-exo1	This work
16	YXB013-84	YXB013-2 + pRS424	This work
17	YXB013-85	YXB013-2 + pRS424-EXO1	This work
18	YXB013-86	YXB013-2 + pRS424-exo1	This work
27	RDKY3615	*MATa ura3-52 leu2Δ1 trp1Δ63 his3Δ200 lys2ΔBgl hom3-10 ade2Δ1 ade8 hxt13::URA3*	Ref. 3
28	YXB013-35	RDKY3615, fun30Δ::NatMX	This work
31	YXB013-37	ZGY005, *rad5Δ::NatMX*	This work
32	YXB013-38	YXB013-37, *fun30Δ::NatMX*	This work
33	YXB013-39	YQY726, *rad5Δ::KanMX*	This work
34	YXB013-40	YXB013-39, *fun30Δ::NatMX*	This work
35	YXB013-41	YQY726, *mms2Δ::KanMX*	This work
36	YXB013-42	YXB013-41, *fun30Δ::NatMX*	This work
37	YXB013-43	YQY726, *ubc13Δ::KanMX*	This work
38	YXB013-44	YXB013-43, *fun30Δ::NatMX*	This work
39	YXB013-45	BY4741, *rad5Δ::KanMX*	Open Biosystems
40	YXB013-46	YXB013-43, *fun30Δ::NatMX*	This work
41	RDKY5519	RDKY3615, *rad5::KanMX*	Ref. 3
42	YXB013-47	RDKY5519, *fun30Δ::NatMX*	This work
43	YXB013-89	YXB013-39, *exo1Δ::NatMX*	This work

44	YXB013-48	YQY726, *rev3Δ::KanMX*	This work
45	YXB013-49	YXB013-48, *fun30Δ::NatMX*	This work
46	YXB013-50	YQY726, *rev7Δ::KanMX*	This work
47	YXB013-51	YXB013-50, *fun30Δ::NatMX*	This work
48	YXB013-52	YQY726, *rad30Δ::KanMX*	This work
49	YXB013-53	YXB013-52, *fun30Δ::NatMX*	This work
50	YXB013-54	YXB013-48, *rad5Δ::ble2*	This work
51	YXB013-55	YXB013-54, *fun30Δ::NatMX*	This work
52	YXB013-56	YXB013-50, *rad5Δ::ble2*	This work
53	YXB013-57	YXB013-56, *fun30Δ::NatMX*	This work
54	YXB013-58	YXB013-52, *rad5Δ::ble2*	This work
55	YXB013-59	YXB013-58, *fun30Δ::NatMX*	This work
56	YXB013-90	YXB013-58, *rev3Δ::TRP1*	This work
57	YXB013-91	YXB013-59, *rev3Δ::TRP1*	This work
58	YXB013-60	YQY726, *mag1Δ::KanMX*	This work
59	YXB013-61	YXB013-60, *fun30Δ::NatMX*	This work
60	YXB013-62	YXB013-60, *rad5Δ::ble2*	This work
61	YXB013-63	YXB013-62, *fun30Δ::NatMX*	This work
62	YXB013-64	YQY726, *rad2Δ::KanMX*	This work
63	YXB013-65	YXB013-64, *rad5Δ::ble2*	This work
64	YXB013-66	YXB013-65, *fun30Δ::NatMX*	This work
65	YXB013-15	YQY726, *yku70Δ::KanMX*	This work
66	YXB013-16	YXB013-15, *fun30Δ::NatMX*	This work
67	YXB013-67	YXB013-15, *rad5Δ::ble2*	This work
68	YXB013-68	YXB013-67, *fun30Δ::NatMX*	This work
69	YXB013-29	YQY726, *rad9Δ::KanMX*	This work
70	YXB013-30	YXB013-29, *fun30Δ::NatMX*	This work
71	YXB013-69	YXB013-29, *rad5Δ::ble2*	This work
72	YXB013-70	YXB013-69, *fun30Δ::NatMX*	This work
73	YXB013-11	YQY726, *rad51Δ::KanMX*	This work
74	YXB013-12	YXB013-11, *fun30Δ::NatMX*	This work
75	YXB013-71	YXB013-11, *rad5Δ::ble2*	This work
76	YXB013-72	YXB013-71, *fun30Δ::NatMX*	This work
77	YXB013-92	YQY726, *pol32Δ::KanMX*	This work
78	YXB013-93	YXB013-92, *fun30Δ::NatMX*	This work
79	YXB013-94	YQY726, *red5Δ::ble2*	This work
80	YXB013-95	YXB013-94, *fun30Δ::NatMX*	This work
81	YXB013-73	YQY726, *srs2Δ::KanMX*	This work
82	YXB013-74	YXB013-73, *fun30Δ::NatMX*	This work
83	YXB013-75	YXB013-73, *rad5Δ::ble2*	This work
84	YXB013-76	YXB013-75, *fun30Δ::NatMX*	This work
85	YXB013-77	YQY726, *siz1Δ::KanMX*	This work
86	YXB013-78	YXB013-77, *fun30Δ::NatMX*	This work
87	YXB013-79	YXB013-77, *rad5Δ::ble2*	This work

88	YXB013-80	YXB013-79, *fun30Δ::NatMX*	This work
89	YXB013-96	YQY726 + pRS414	This work
90	YXB013-97	YQY726 + pRS414-FUN30	This work
91	YXB013-98	YQY726 + pRS424-FUN30-CUEΔ	This work
92	YXB013-99	YQY726 + pRS424-FUN30-CUE**	This work
93	YXB013-100	YQY726 + pRS424-FUN30-K603R	This work
94	YXB013-101	YXB013-2 + pRS414	This work
95	YXB013-102	YXB013-2 + pRS414-FUN30	This work
96	YXB013-103	YXB013-2 + pRS424-FUN30-CUEΔ	This work
97	YXB013-104	YXB013-2 + pRS424-FUN30-CUE**	This work
98	YXB013-105	YXB013-2 + pRS424-FUN30-K603R	This work
99	RDKY6649	RDKY3615, *POL30-His*$_6$*-FLAG::KanMX*	Ref. 3
100	YXB013-106	RDKY6649, *fun30Δ::NatMX*	This work

References

1. Roy N, Runge KW. Two paralogs involved in transcriptional silencing that antagonistically control yeast life span. Curr Biol. 2000;10: 111-114.

2. Zhang Z, Shibahara K, Stillman B. PCNA connects DNA replication to epigenetic inheritance in yeast. Nature. 2000;408: 221-225.

3. Kats ES, Enserink JM, Martinez S, Kolodner RD. The *Saccharomyces cerevisiae* Rad6 postreplication repair and Siz1/Srs2 homologous recombination-inhibiting pathways process DNA damage that arises in *asf1* mutants. Mol Cell Biol. 2009;29: 5226-5237.
